# Inflammatory cytokines and aromatase inhibitor-associated musculoskeletal syndrome: a case–control study

**DOI:** 10.1038/sj.bjc.6605768

**Published:** 2010-07-06

**Authors:** N L Henry, D Pchejetski, R A'Hern, A T Nguyen, P Charles, J Waxman, L Li, A M Storniolo, D F Hayes, D A Flockhart, V Stearns, J Stebbing

**Affiliations:** 1 Division of Hematology/Oncology, Department of Internal Medicine, University of Michigan Medical School, 300 North Ingalls, North Ingalls Building 3A04, Ann Arbor, MI 48109-5419, USA; 2 Department of Oncology, Imperial College Healthcare NHS Trust, 501 Cyclotron Building, Hammersmith hospital, Ducane Road, London W120NN, UK; 3 Cancer Research UK Clinical Trials and Statistics Unit, The Institute of Cancer Research, Sir Richard Doll Building, Cotswold Road, Sutton, Surrey SM2 5NG, UK; 4 Division of Clinical Pharmacology, Department of Medicine, Indiana University School of Medicine, 1001 West 10th Street, Indianapolis, IN 46202, USA; 5 Kennedy Institute of Rheumatology, Imperial College, Aspenlea Road, London W6 8RF, UK; 6 Department of Medicine, Division of Hematology/Oncology, Melvin and Bren Simon Cancer Center at Indiana University, Indianapolis, IN 46202, USA; 7 Department of Oncology, Breast Cancer Program, Sidney Kimmel Comprehensive Cancer Center, Johns Hopkins School of Medicine, Bunting-Blaustein Cancer Research Building, 1650 Orleans Street, Room 145, Baltimore, MD 21231-1000, USA

**Keywords:** aromatase inhibitor, arthralgia, breast cancer, cytokine

## Abstract

**Background:**

The aromatase inhibitor (AI)-associated musculoskeletal syndrome (AIMSS) occurs in approximately 50% of AI-treated patients. Inflammatory mediators are associated with oestrogen signalling and may change with oestrogen depletion. We hypothesised that AIMSS may be associated with changes in circulating inflammatory markers.

**Methods:**

Patients with breast cancer were enroled in a trial of adjuvant AI therapy. Changes in pain and function during therapy were assessed prospectively. We selected 30 cases with AIMSS and 22 controls without AIMSS, matched for demographics and prior therapy. Serum samples collected at baseline and during treatment were assayed for multiple inflammatory cytokines and lipid mediators using multiplex assays.

**Results:**

Before AI therapy, mean serum concentrations of 6 of 36 assayed factors were statistically significantly lower in cases than controls (all *P*<0.003). No statistically significant changes during AI therapy relative to pre-treatment were observed between cases and controls for any of the inflammatory markers tested.

**Conclusion:**

AIMSS is probably not associated with a systemic inflammatory response. Pre-treatment cytokine levels may predict for development of AIMSS.

Adjuvant endocrine therapy decreases mortality for women with invasive hormone receptor (HR)-positive breast cancer (Peto *et al*, 2000; EBCTCG, 2005). The three third-generation aromatase inhibitors (AIs) used in routine clinical practice in post-menopausal women (anastrozole, letrozole, and exemestane; Boccardo *et al*, 2005; Goss *et al*, 2005; Jakesz *et al*, 2005; Winer *et al*, 2005; Coates *et al*, 2007; Coombes *et al*, 2007; Forbes *et al*, 2008; Mamounas *et al*, 2008) act by inhibiting the aromatase enzyme, thereby substantially decreasing serum oestrogen concentrations (Geisler *et al*, 2002).

Aromatase inhibitor-associated musculoskeletal symptoms (AIMSS), including arthralgias and myalgias, have been noted in up to 50% of treated patients (Burstein and Winer, 2007; Crew *et al*, 2007; Henry *et al*, 2008a, 2008b). Severe toxicity resulted in a greater than 10% treatment discontinuation rate in a recently reported trial (Henry *et al*, 2008a). A variety of pharmacological interventions to prevent or treat AIMSS have been largely ineffective (Burstein and Winer, 2007; Crew *et al*, 2007; Henry *et al*, 2008a, 2008b). However, recent data from a large randomised controlled trial demonstrated that development of AI-associated joint pain might predict improved breast cancer outcomes (Cuzick *et al*, 2008).

The aetiology of AIMSS remains undetermined. Clinical data have not supported the hypothesis that a systemic inflammatory process causes AIMSS (Henry *et al*, 2008b). However, imaging studies have demonstrated the development of tenosynovitis in patients treated with AIs (Morales *et al*, 2008), suggesting that localised inflammation may occur.

Oestrogen is known to have a role in the immune system (Carlsten, 2005). Inflammatory mediators, including interleukins (ILs), interferons, and matrix metalloproteinases, have been associated with changes in oestrogenic signalling (Straub, 2007). Increases in pro-inflammatory cytokine levels, including those of IL-1, tumour necrosis factor-alpha, and IL-6, can occur during menopause (Pfeilschifter *et al*, 2002). Recent data have also demonstrated that lipid mediators may influence the action of oestrogens (Sukocheva *et al*, 2009). Thus, multiple oestrogen-dependent inflammatory mediators may be associated with the development of a localised inflammatory process.

We therefore hypothesised that AIMSS may be associated with alterations in serum concentrations of pro-inflammatory mediators. To test this hypothesis, we evaluated changes in serum concentrations of multiple inflammatory molecules in subjects enroled in a multicenter prospective clinical trial of AI therapy

## Materials and methods

This is a case–control study embedded in a larger prospective randomised controlled trial designated ELPh (Exemestane and Letrozole Pharmacogenetics, NCT00228956), which has been previously described in detail (Henry *et al*, 2008a).

### Subjects

From August 2005 to October 2007, post-menopausal women with stage 0–III breast cancer were recruited at the following Consortium on Breast Cancer Pharmacogenomics (COBRA) institutions: Indiana University Cancer Center, Sidney Kimmel Comprehensive Cancer Center at The Johns Hopkins School of Medicine, and the University of Michigan Comprehensive Cancer Center. Subjects were ineligible if they had received prior AI therapy. Prior tamoxifen and concurrent trastuzumab treatments were permitted. All indicated surgery, radiation therapy, and chemotherapy for breast cancer was completed before study enrolment. The protocol was approved by the Institutional Review Boards of all three participating study sites.

### Study design

After providing written informed consent, subjects were randomly assigned to two treatment groups: 25 mg exemestane orally daily or 2.5 mg letrozole orally daily for 2 years. At baseline and at 1, 3, 6, and 12 months after AI therapy initiation, serum samples were collected and subjects completed the two-page Health Assessment Questionnaire and pain Visual Analog Scale (Bruce and Fries, 2003), a well-validated tool that has been used to evaluate the functional status and pain of patients with rheumatic disorders. Patients were referred for a detailed evaluation by a designated rheumatologist at each site if they met pre-defined criteria for increase in pain or decrease in functional status based on scores on questionnaires (Henry *et al*, 2008a). In brief, subjects met criteria for referral if they had (1) an increase in Health Assessment Questionnaire score of >0.4 if their baseline score was 0–0.8; (2) an increase in Health Assessment Questionnaire score of >0.2 if their baseline score was 0.8–1.5; (3) a pain Visual Analog Scale score above 5 if they had no pain at baseline; and/or (4) ‘much worse’ or ‘very much worse’ pain based on the Self-rated Clinical Global Impression Change Scale if they reported pain at the time of AI initiation. Treating oncologists could also refer a subject for rheumatologic evaluation if they felt that the subject had AI-associated musculoskeletal symptoms even if the subject did not meet the pre-defined criteria. At the time of evaluation, the rheumatologists reported the symptom severity and impact on patient function, as well as the apparent association between symptoms and AI therapy (probably, possibly, or not related).

### Selection of cases and controls

This hypothesis-generating case–control study was conducted using samples and data from the ELPh trial. At the time of selection of cases and controls, 260 subjects had completed 12 months of protocol-directed therapy. Cases were selected by the coordinating center from those subjects who met pre-defined criteria for rheumatologic evaluation within 12 months of AI initiation and who were understood to have AI-associated symptoms by the evaluating rheumatologist or treating oncologist. Controls, defined as those subjects who did not meet criteria for rheumatologic evaluation during the first 12 months of AI therapy, were matched to the cases based on AI medication, age (±5 years), prior chemotherapy (yes/no), prior tamoxifen (yes/no), and ethnicity.

### Quantification of serum inflammatory markers

We selected serum markers for analysis that had previously been implicated in inflammatory responses, pain, arthritis, and menopause (Pfeilschifter *et al*, 2002; Sommer and Kress, 2004; Brennan and McInnes, 2008; Sukocheva *et al*, 2009). Serum samples from the ELPh trial were stored at −70°C until analysed; all samples were assayed simultaneously in a blinded manner according to manufacturer's instructions. The inflammatory markers listed in Supplementary Table 1 were assayed. Cytokine and matrix metalloproteinase levels were measured using an addressable laser bead multiplex assay (BMD, Paris, France) on a Luminex S100 analyser (Luminex Corporation, Austin, TX, USA); multiple proteins were detected simultaneously within a serum sample (Stebbing *et al*, 2008; Bower *et al*, 2009). The results were calculated using StarStation 2.0 software system (Applied Cytometry Systems, Sheffield, UK). Ceramide and sphingosine-1-phosphate were extracted from serum and quantified as previously described (Folch *et al*, 1957; Liu *et al*, 2000; Bonhoure *et al*, 2006).

### Statistical analyses

Statistical comparisons for each factor were performed by comparing cases with controls. This approach maximised the number of subjects in the analyses by allowing unpaired cases and controls to be included. The non-parametric Mann–Whitney *U*-test was used to compare case and control values for categorical data, and the unpaired two-tailed *t*-test was used for continuous data.

For continuous data, values were log-transformed before analysis because they were frequently observed to be positively skewed. This implies that proportional values are then the subject of the analyses when changes are being examined, for example, log(1 month value)–log(baseline value)=log(1 month value/baseline value). Results were summarised by back-transforming the means and confidence interval values obtained from the analyses on the log-transformed data. Continuous data were analysed as categorical variables if a significant proportion of values fell below the lower limit of detection for the individual assay. Changes from baseline to 1 and 6 months were categorised into four groups: an increase in score; no change in score; a decrease but not below the limit of detection; and a decrease below the limit of detection. These four categories were scored 1–4 and this score was used for non-parametric statistical comparisons.

Subsequently, statistical comparisons were performed taking the case and control pairings into account. The Wilcoxon signed rank test was used for categorical data and the paired *t*-test was used for continuous data.

All *P*-values are uncorrected for multiple comparisons. As this was an exploratory study, a false discovery rate correction was used. This approach is more appropriate to allow for multiple testing rather than a family-wise error rate correction, such as a Bonferroni correction. The former is less stringent and controls the proportion of false positives (in this case 5%) in the tests declared significant, rather than ensuring the probability that there are *any* tests at all declared falsely significant is 5%. The uncorrected *P*-value that corresponds to a false discovery rate of 5% in this study was *P*=0.003.

## Results

### Patient characteristics

We selected 30 cases who enroled in the prospective ELPh trial and who were referred for rheumatological evaluation because of increased pain or worsened functional status during AI therapy. One case did not meet the pre-defined criteria for referral based on questionnaire data, but was diagnosed with AI-associated musculoskeletal symptoms by her treating oncologist. Thirty matched controls, who did not experience increased pain or worsened functional status during AI therapy, were then selected. However, eight controls were subsequently deemed ineligible because they failed to complete 12 months of AI treatment for reasons unrelated to musculoskeletal symptoms and therefore were not included in any analyses. Baseline characteristics of the cases and included controls are presented in Table 1. Approximately half of cases and half of controls reported taking non-steroidal medications during study participation. Of the cases, 17 of 30 prematurely discontinued study participation a median of 7.2 months (range 2–19 mo) after enrolment because of arthralgias (*n*=10), carpal tunnel syndrome (*n*=3), myalgias (*n*=2), or fatigue/insomnia (*n*=2).

There was no statistically significant difference in pain severity or functional status between cases and controls before the initiation of AI therapy (data not shown). Pain severity was statistically significantly different between cases and controls at 1, 3, and 6 months after treatment initiation. Functional status as assessed using the Health Assessment Questionnaire instrument was statistically significantly different between the two groups only at the 6-month time point.

### Baseline serum concentrations of multiple inflammatory markers

**Figure 1:**
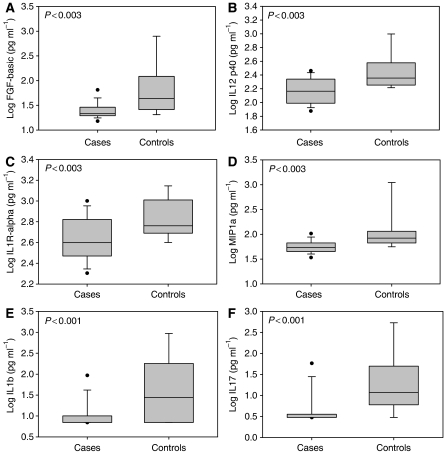
Baseline serum concentrations of selected inflammatory markers. (**A**) Fibroblast growth factor-basic; (**B**) interleukin-12 p40; (**C**) interleukin-1 receptor *α*; (**D**) macrophage inflammatory protein-1; (**E**) interleukin-1b; (**F**) interleukin-17 for cases compared with controls. Data are presented as box plots of the log of geometric means for continuous data (**A**–**D**), and log of medians for data with highly skewed distributions arising because of lower detectability limits (**E** and **F**). Cases: *n*=30. Controls: *n*=18.

Serum concentrations of baseline inflammatory markers from cases were compared with those of controls. Not all samples were assayed for all factors due to insufficient quantity of serum. Baseline serum concentrations of fibroblast growth factor-basic, IL-1 receptor-*α*, IL-12 p40, macrophage inflammatory protein-1*α*, IL-1b, and IL-17 were statistically significantly lower in cases than in controls (all *P*<0.003; Figure 1). At baseline, the mean serum concentration of only one inflammatory marker, RANTES (regulated upon activation, normal T cell expressed and secreted), was higher in cases compared with controls (18 344 *vs* 15 721 pg ml^−1^; *P*=0.03) although the difference was not statistically significant when adjusted to avoid false discovery (see Materials and Methods section). Results for all assayed cytokines are provided in Supplementary Table 2.

Baseline serum concentrations of inflammatory factors were also analysed in matched pairs of cases and controls. Similar to the comparison of all cases *vs* all controls, there was a statistically significant difference between matched cases and controls for IL-17, IL-12 p40, and IP-10 (all *P*<0.003). In contrast, two factors were present at higher concentrations in cases compared with controls, although the differences were not statistically significant when adjusted to avoid false discovery. The ratio of matched cases to controls for RANTES was 1.22 (0.91–1.63; *P*=0.01), and for matrix metalloproteinase-9 was 1.92 (1.16–3.17, *P*=0.01). Levels of all other assayed factors were unchanged or were higher in matched controls compared with cases, but the differences were not statistically significant (data not shown).

### Change in serum inflammatory markers during AI therapy

Changes in serum inflammatory marker concentrations during AI therapy relative to pre-treatment baseline were assessed. Not all subjects had serum available at all time points, including those who discontinued AI therapy before the 6-month evaluation. A non-significant trend towards increased lipid mediator S1P concentrations in cases (+13%) and decreased (−39%) S1P in controls was observed at the 6-month time point compared with baseline (*P*=0.08). There was also a trend towards a greater percentage of cases with decreased serum concentrations of IL-6 at the 6-month time point (54.2%) compared with controls (25% *P*=0.08). Overall, there were no statistically significant differences in change in serum concentration of any inflammatory markers during AI therapy when all cases were compared with all controls (data for all cytokines at 1 and 6 months are provided in Supplementary Tables 3 and 4) or when matched cases and controls were analysed (data not shown).

## Discussion

Musculoskeletal symptoms are increasingly recognised to be a clinically significant toxicity of AI therapy, but the mechanism underlying development of these symptoms remains unclear. As a result, no clinical predictors of toxicity development have been identified. In this case–control study, using samples from a prospective trial, we describe differences in multiple pleiotropic serum inflammatory factors in post-menopausal women who develop this toxicity compared with those who remain asymptomatic.

In contrast to our original hypothesis, in our study we observed few differences in serum concentrations of inflammatory markers during AI therapy between cases and controls, suggesting that AIMSS is not due to a systemic inflammatory response. In addition, the trend towards decreased IL-6 levels in affected subjects, which is in contrast to published reports of the role of IL-6 in inflammation (Choy and Panayi, 2001; Gabay, 2006), further supports the lack of an inflammatory mechanism underlying AIMSS. These data are consistent with previously reported findings of lack of anti-nuclear antibodies and/or elevated erythrocyte sedimentation rate in affected patients (Azria *et al*, 2006; Henry *et al*, 2008a), but in contrast with results from a prospective study of 24 patients with significant AI-associated pain and concomitant elevations in anti-nuclear antibodies and rheumatoid factor (Laroche *et al*, 2007).

Although no difference in cytokines during AI treatment was detected between cases and controls, subjects who developed AIMSS had lower concentrations of multiple cytokines before treatment initiation, including both pro- and anti-inflammatory molecules. This finding was unexpected. It is unlikely to be due to prior chemotherapy or tamoxifen, or to specific AI medication, as cases and controls were matched for both prior treatment regimen and assigned AI. In addition, the pattern seems different from that observed in other systemic inflammatory processes, such as rheumatoid arthritis, tuberculosis, sarcoidosis, and multi-system disorders such as multicentric Castleman's disease (Bower *et al*, 2007, 2009; Stebbing *et al*, 2008).

Patients with metastatic breast cancer treated with AI therapy seldom report these musculoskeletal symptoms. One report noted 16% of anastrozole-treated patients with metastatic breast cancer developed arthralgias, although only a minority developed severe symptoms (Donnellan *et al*, 2001). One hypothesis is that patients with ‘active cancer’ may have higher circulating levels of cytokines before initiation of therapy, and therefore be less susceptible to development of symptoms. Alternatively, less emphasis may be placed on these symptoms in patients with other cancer-related symptoms.

One limitation of this study is the focus on systemic as opposed to local concentrations of inflammatory factors. Others have suggested that local inflammatory processes may be occurring, such as tenosynovitis at the wrist, as demonstrated using magnetic resonance imaging (Morales *et al*, 2008). Therefore, future mechanistic studies could be designed to investigate changes at the local level.

Another limitation of this study is the small sample size and large number of analysed factors. In addition, although it is not unexpected that subjects may have to discontinue AI therapy prematurely because of the development of AIMSS (Henry *et al*, 2008a), a number of controls also discontinued therapy before the 12-month time point for reasons unrelated to arthralgias and had to be excluded from this analysis, as it was unknown whether they would have developed AIMSS had they continued study participation for the entire duration. This further limited statistical power.

In summary, contrary to our initial hypothesis, we failed to observe an association between AIMSS and a systemic inflammatory response after initiation of an AI. Therefore, if oestrogen deprivation is the underlying cause of this musculoskeletal toxicity, it may be inducing a localised inflammatory effect, such as in the bone or tendon, rather than a global effect, such as the increase in systemic inflammatory factors seen at the time of menopause. Alternatively, AIMSS could be due to a non-inflammatory process. Overall, these data suggest that AIMSS is distinct from other rheumatologic conditions, and continued research into aetiologies and management approaches is warranted.

## Figures and Tables

**Table 1:** Subject characteristics

**Characteristic**	**Cases (*n*=30)**	**Controls (*n*=22)**
Median age (years, range)	56.9 (47–67)	58.4 (50–71)
Median weight (kg, range)	81.5 (55.8–150.6)	77.6 (57–108)
Median BMI (kg m^−2^, range)	30.5 (22.3–48.5)	29.2 (21.0–38.7)
Prior chemotherapy	17 (56.7%)	13 (59.1%)
Prior taxane treatment	10 (34.5%)^a^	7 (31.8%)
Prior tamoxifen treatment	15 (50%)	9 (40.9%)

Abbreviation: BMI=body mass index.

a One unknown.
