# Supplementary material for: Inflammatory cytokines and aromatase inhibitor-associated musculoskeletal syndrome: a case–control study
Source: Br J Cancer. 2010 Jul 6;103(3):291–6. doi: 10.1038/sj.bjc.6605768 (PMC2920022; doi:10.1038/sj.bjc.6605768)
Supplement: Supplementary Table 1 [file 6605768x1.doc]

**Supplemental Table 1.** The following inflammatory markers were evaluated:

Inflammatory mediators

Epidermal growth factor (EGF)

Eotaxin

Fibroblast growth factor (FGF-basic)

Granulocyte-Colony stimulating factor (G-CSF)

Granulocyte-Macrophage-Colony stimulating factor (GM-CSF)

Human growth factor (HGF)

Interferon alpha (IFNα)

Interferon gamma (IFNγ)

Interleukin-1β (IL1β)

Interleukin-1 receptor alpha (IL1Rα)

Interleukin-2 (IL2)

Interleukin-2 receptor (IL2R)

Interleukin-4 (IL4)

Interleukin-5 (IL5)

Interleukin-6 (IL6)

Interleukin-7 (IL7)

Interleukin-8 (IL8)

Interleukin-10 (IL10)

Interleukin-12 p40 (IL12 p40)

Interleukin-13 (IL13)

Interleukin-15 (IL15)

Interleukin-17 (IL17)

Interferon-inducible protein 10 (IP10)

Monocyte chemoattractant protein 1 (MCP1)

Monokine induced by gamma interferon (MIG)

Macrophage Inflammatory Protein 1 alpha (MIP1α)

Macrophage Inflammatory Protein 1 beta (MIP1β)

RANTES/CCL5

Tumor necrosis factor-receptor I (TNF-RI)

Tumor necrosis factor-receptor II (TNF-RII)

Vascular endothelial growth factor (VEGF)

Matrix metalloproteinases (MMP)

MMP-3

MMP-9

MMP-13

Lipid mediators

Ceramide

Sphingosine-1-phosphate (S1P)
