# Supplementary material for: Inflammatory cytokines and aromatase inhibitor-associated musculoskeletal syndrome: a case–control study
Source: Br J Cancer. 2010 Jul 6;103(3):291–6. doi: 10.1038/sj.bjc.6605768 (PMC2920022; doi:10.1038/sj.bjc.6605768)
Supplement: Supplementary Table 2 [file 6605768x2.doc]

**Supplemental** **Table 2.** Baseline serum concentrations of inflammatory markers for cases compared to controls. A. Geometric means and 95% confidence intervals for continuous data. B. Medians and 95% confidence intervals for data with highly skewed distributions arising as a result of lower detectability limits. N = number of subjects. A p-value of 0.003 or less corresponds to a false discovery rate of 5%.

**A.**

| Marker | Cases | Controls | P value |
| --- | --- | --- | --- |
| Eotaxin (pg/ml) | 64 (51.4-78.4)  n=30 | 75.4 (52.9-107.2)  n=18 | 0.16 |
| FGF-basic (pg/ml) | 24.8 (19.3-31.2)  n=30 | 63.1 (36.8-108.4)  n=18 | <0.003 |
| G-CSF (pg/ml) | 65.8 (47.7-88.6)  n=30 | 167.4 (89.1-314.4)  n=18 | 0.01 |
| HGF (pg/ml) | 381.5 (288.8-494.3)  n=30 | 506.8 (344.5-745.7)  n=18 | 0.08 |
| IFNa (pg/ml) | 23.4 (18.6-28.9)  n=30 | 35.5 (20.7-61.1)  n=18 | 0.07 |
| IL1Ra (pg/ml) | 430.8 (324.6-560.7)  n=30 | 929.9 (552-1566.4)  n=18 | <0.003 |
| IL2R (pg/ml) | 194.3 (151.2-245.3)  n=30 | 292.6 (185.7-461)  n=18 | 0.03 |
| IL7 (pg/ml) | 88.4 (71.5-107.8)  n=30 | 110.3 (85.2-142.8)  n=18 | 0.01 |
| IL8 (pg/ml) | 9.1 (6-13.4)  n=30 | 9.7 (5.6-16.9)  n=18 | 0.84 |
| IL12 p40 (pg/ml) | 147.5 (112.9-189.1)  n=30 | 280.2 (186.9-420)  n=18 | <0.003 |
| IP10 (pg/ml) | 15.8 (12.1-20.2)  n=30 | 23.3 (15.9-34.1)  n=18 | 0.01 |
| MCP1 (pg/ml) | 453.6 (359.6-563.1)  n=30 | 518.5 (330.1-814.6)  n=18 | 0.43 |
| MIP1a (pg/ml) | 55.2 (44.2-67.8)  n=30 | 113.1 (69.2-184.8)  n=18 | <0.003 |
| MIP1b (pg/ml) | 46.2 (34.3-61)  n=30 | 75.3 (46-123.3)  n=18 | 0.03 |
| RANTES (pg/ml) | 18344 (14910-22249)  n=30 | 15721 (12896-19163)  n=18 | 0.03 |
| MMP-3 (pg/ml) | 3950.7 (3075.3-5008.1)  n=29 | 3765.1 (2752.7-5150)  n=22 | 0.7 |
| MMP-9 (pg/ml) | 136559 (88780-205300), n=29 | 96332 (68934-134619), n=22 | 0.2 |
| Marker | Cases | Controls | P value |
| MMP-13 (pg/ml) | 193.5 (135.9-270.5)  n=29 | 240.8 (156.2-371.4)  n=22 | 0.37 |
| pmol S1P/ug protein in serum | 5.3 (3.7-7.6)  n=29 | 4.8 (3.1-7.3)  n=21 | 0.65 |
| pmol ceramide/ug protein in serum | 2 (1.6-2.6)  n=29 | 2.4 (1.9-3.1)  n=21 | 0.09 |
| TNF-RI (pg/ml) | 1558.6 (1230.7-1942)  n=30 | 1668.4 (1172.4-2374.2)  n=19 | 0.59 |
| TNF-RII (pg/ml) | 1614.8 (1169.6-2180.3)  n=30 | 2061.8 (1539-2762.4)  n=19 | 0.14 |

B.

| Marker | Cases | Controls | P value |
| --- | --- | --- | --- |
| EGF (pg/ml) | 18.9 (10.7-34.4)  n=30 | 20.1 (5.1-63.6)  n=18 | 0.78 |
| GM-CSF (pg/ml) | 4 (4-12)  n=30 | 24 (8-223.9)  n=18 | 0.004 |
| IFNg (pg/ml) | 4 (4-6.5)  n=30 | 6.7 (4-12)  n=18 | 0.12 |
| IL1b (pg/ml) | 7 (7-7)  n=30 | 27.5 (7-174.5)  n=18 | <0.001 |
| IL2 (pg/ml) | 2 (2-2)  n=30 | 3.5 (2-28)  n=18 | 0.02 |
| IL4 (pg/ml) | 8 (6-12)  n=30 | 13.4 (7.2-20.2)  n=18 | 0.11 |
| IL5 (pg/ml) | 1 (1-1.62)  n=30 | 1.6 (1-3.2)  n=18 | 0.13 |
| IL6 (pg/ml) | 2.1 (1.2-3.2)  n=30 | 2.7 (1.2-8.5)  n=16 | 0.34 |
| IL10 (pg/ml) | 1 (1-1.12)  n=30 | 1.7 (1-17.7)  n=18 | 0.007 |
| IL13 (pg/ml) | 4 (2-5.5)  n=31 | 5.0 (2-8.3)  n=18 | 0.13 |
| IL15 (pg/ml) | 8.3 (7. 7-11)  n=30 | 12.4 (9.342-41.1)  n=18 | 0.007 |
| IL17 (pg/ml) | 3 (3-3)  n=30 | 11.8 (6-41.6)  n=18 | <0.001 |
| MIG (pg/ml) | 26.0 (16-33.2)  n=30 | 46 (26-81.2)  n=18 | 0.05 |
| VEGF (pg/ml) | 4.9 (3-10.2)  n=30 | 14.9 (3-38.4)  n=18 | 0.37 |
