# Supplementary material for: Inflammatory cytokines and aromatase inhibitor-associated musculoskeletal syndrome: a case–control study
Source: Br J Cancer. 2010 Jul 6;103(3):291–6. doi: 10.1038/sj.bjc.6605768 (PMC2920022; doi:10.1038/sj.bjc.6605768)
Supplement: Supplementary Table 3 [file 6605768x3.doc]

**Supplemental** **Table 3.** Change in serum concentrations of inflammatory markers at 1 or 6 months compared to baseline for cases compared to controls. Value given is ratio of serum concentration at indicated timepoint to baseline. N = number of subjects. A p-value of 0.003 or less corresponds to a false discovery rate of 5%.

| **Marker** | **Timepoint** | **Cases** | **Controls** | **P value** |
| --- | --- | --- | --- | --- |
| Eotaxin | 1 mo | 0.96 (0.74-1.24)  n=26 | 1.16 (0.74-1.83)  n=12 | 0.23 |
|  | 6 mo | 1.04 (0.78-1.39)  n=24 | 1.01 (0.75-1.36)  n=14 | 0.85 |
| FGF-basic | 1 mo | 0.95 (0.73-1.22)  n=26 | 1.17 (0.72-1.91)  n=12 | 0.20 |
|  | 6 mo | 0.96 (0.78-1.18)  n=24 | 0.97 (0.68-1.38)  n=14 | 0.93 |
| G-CSF | 1 mo | 0.88 (0.63-1.23)  n=26 | 1.31 (0.77-2.25)  n=12 | 0.11 |
|  | 6 mo | 0.89 (0.67-1.2)  n=24 | 0.87 (0.58-1.31)  n=14 | 0.87 |
| HGF | 1 mo | 0.92 (0.71-1.19)  n=26 | 1.01 (0.73-1.41)  n=12 | 0.46 |
|  | 6 mo | 0.96 (0.76-1.21)  n=24 | 1.00 (0.76-1.33)  n=14 | 0.62 |
| IFNa | 1 mo | 0.93 (0.73-1.18)  n=26 | 1.13 (0.67-1.90)  n=12 | 0.25 |
|  | 6 mo | 0.95 (0.78-1.17)  n=24 | 0.88 (0.64-1.21)  n=14 | 0.37 |
| IL1Ra | 1 mo | 1.03 (0.79-1.34)  n=26 | 1.15 (0.75-1.76)  n=12 | 0.46 |
|  | 6 mo | 1.03 (0.79-1.35)  n=24 | 0.98 (0.67-1.42)  n=14 | 0.71 |
| IL2R | 1 mo | 0.97 (0.74-1.28)  n=26 | 0.99 (0.71-1.38)  n=12 | 0.88 |
|  | 6 mo | 1.02 (0.79-1.32)  n=24 | 0.94 (0.69-1.26)  n=14 | 0.44 |
| IL7 | 1 mo | 0.99 (0.78-1.26)  n=26 | 0.98 (0.71-1.36)  n=12 | 0.93 |
|  | 6 mo | 0.94 (0.78-1.13)  n=24 | 0.95 (0.74-1.23)  n=14 | 0.84 |
| IL8 | 1 mo | 1.33 (0.95-1.86)  n=26 | 1.05 (0.57-1.93)  n=12 | 0.38 |
|  | 6 mo | 0.90 (0.60-1.37)  n=24 | 1.05 (0.64-1.71)  n=14 | 0.63 |

| **Marker** | **Timepoint** | **Cases** | **Controls** | **P value** |
| --- | --- | --- | --- | --- |
| IL12 p40 | 1 mo | 0.98 (0.77-1.24)  n=26 | 1.12 (0.77-1.62)  n=12 | 0.28 |
|  | 6 mo | 1.05 (0.82-1.33)  n=24 | 1.02 (0.73-1.41)  n=14 | 0.80 |
| IP10 | 1 mo | 1.01 (0.78-1.31)  n=26 | 0.88 (0.60-1.30)  n=12 | 0.33 |
|  | 6 mo | 1.00 (0.75-1.33)  n=24 | 0.84 (0.57-1.23)  n=14 | 0.29 |
| MCP1 | 1 mo | 1.03 (0.79-1.34)  n=26 | 1.09 (0.68-1.76)  n=12 | 0.71 |
|  | 6 mo | 1.01 (0.75-1.35)  n=24 | 1.04 (0.78-1.39)  n=14 | 0.81 |
| MIP1a | 1 mo | 0.97 (0.77-1.24)  n=26 | 0.98 (0.72-1.32)  n=12 | 0.95 |
|  | 6 mo | 0.92 (0.76-1.11)  n=24 | 0.99 (0.71-1.39)  n=13 | 0.35 |
| MIP1b | 1 mo | 0.92 (0.70-1.21)  n=26 | 1.04 (0.68-1.58)  n=12 | 0.47 |
|  | 6 mo | 0.98 (0.75-1.26)  n=24 | 1.00 (0.69-1.46)  n=13 | 0.82 |
| RANTES | 1 mo | 1.04 (0.80-1.34)  n=26 | 0.98 (0.77-1.25)  n=12 | 0.64 |
|  | 6 mo | 1.05 (0.85-1.29)  n=24 | 0.94 (0.69-1.27)  n=13 | 0.19 |
| MMP-3 | 1 mo | 1.05 (0.81-1.36)  n=25 | 1.19 (0.91-1.55)  n=16 | 0.29 |
|  | 6 mo | 1.00 (0.78-1.28)  n=23 | 1.13 (0.85-1.50)  n=15 | 0.24 |
| MMP-9 | 1 mo | 0.74 (0.47-1.17)  n=25 | 1.02 (0.73-1.43)  n=16 | 0.19 |
|  | 6 mo | 1.43 (0.87-2.35)  n=23 | 1.12 (0.83-1.51)  n=15 | 0.48 |
| MMP-13 | 1 mo | 0.90 (0.64-1.27)  n=25 | 1.23 (0.76-1.98)  n=16 | 0.20 |
|  | 6 mo | 1.05 (0.76-1.46)  n=23 | 1.28 (0.89-1.84)  n=15 | 0.26 |
| S1P | 1 mo | 0.92 (0.59-1.44)  n=25 | 1.04 (0.59-1.85)  n=13 | 0.74 |
|  | 6 mo | 1.13 (0.73-1.77)  n=23 | 0.61 (0.35-1.05)  n=16 | 0.08 |

| **Marker** | **Timepoint** | **Cases** | **Controls** | **P value** |
| --- | --- | --- | --- | --- |
| ceramide | 1 mo | 1.03 (0.75-1.41)  n=25 | 1.06 (0.80-1.39)  n=13 | 0.87 |
|  | 6 mo | 1.01 (0.70-1.46)  n=23 | 1.02 (0.78-1.33)  n=16 | 0.97 |
| TNF RI | 1 mo | 1.01 (0.82-1.24)  n=26 | 1.17 (0.82-1.66)  n=13 | 0.16 |
|  | 6 mo | 0.99 (0.82-1.20)  n=24 | 1.16 (0.84-1.60)  n=15 | 0.08 |
| TNF RII | 1 mo | 1.02 (0.86-1.20)  n=26 | 1.06 (0.80-1.40)  n=13 | 0.52 |
|  | 6 mo | 1.06 (0.85-1.33)  n=24 | 0.95 (0.71-1.27)  n=15 | 0.24 |
