# Supplementary material for: Inflammatory cytokines and aromatase inhibitor-associated musculoskeletal syndrome: a case–control study
Source: Br J Cancer. 2010 Jul 6;103(3):291–6. doi: 10.1038/sj.bjc.6605768 (PMC2920022; doi:10.1038/sj.bjc.6605768)
Supplement: Supplementary Table 4 [file 6605768x4.doc]

**Supplemental** **Table 4.** Percentage of cases versus controls with an increase or decrease in serum concentrations of inflammatory markers at 1 or 6 months relative to baseline. N = number of subjects. P value reflects overall comparison of percentage of values that increase, remain stable, or decrease between cases and controls for each marker at each timepoint. A p-value of 0.003 or less corresponds to a false discovery rate of 5%.

| **Marker** | **Timepoint** |  | **# subjects** | **Increase** | **Decrease** | **P value** |
| --- | --- | --- | --- | --- | --- | --- |
| EGF | 1 mo | Cases | n=27 | 18.5% | 55.5% | 0.13 |
|  |  | Controls | n=12 | 33.3% | 50% |  |
|  | 6 mo | Cases | n=24 | 33.3% | 50% | 0.26 |
|  |  | Controls | n=14 | 42.9% | 35.7% |  |
| GM-CSF | 1 mo | Cases | n=26 | 26.9% | 23% | 0.25 |
|  |  | Controls | n=12 | 16.7% | 41.6% |  |
|  | 6 mo | Cases | n=24 | 16.7% | 37.5% | 0.37 |
|  |  | Controls | n=14 | 35.7% | 35.7% |  |
| IFN gamma | 1 mo | Cases | n=26 | 11.5% | 30.7% | 0.46 |
|  |  | Controls | n=12 | 16.7% | 25% |  |
|  | 6 mo | Cases | n=24 | 8.3% | 25% | 0.52 |
|  |  | Controls | n=14 | 28.6% | 28.5% |  |
| IL-1b | 1 mo | Cases | n=26 | 15.4% | 19.2% | 0.30 |
|  |  | Controls | n=12 | 41.7% | 25% |  |
|  | 6 mo | Cases | n=24 | 4.2% | 25% | 0.97 |
|  |  | Controls | n=14 | 28.6% | 50% |  |
| IL-2 | 1 mo | Cases | n=26 | 11.5% | 19.2% | 0.20 |
|  |  | Controls | n=12 | 41.7% | 25% |  |
|  | 6 mo | Cases | n=24 | 4.2% | 16.7% | 0.89 |
|  |  | Controls | n=14 | 28.6% | 35.7% |  |
| IL-4 | 1 mo | Cases | n=26 | 15.4% | 61.5% | 0.13 |
|  |  | Controls | n=12 | 41.7% | 50% |  |
|  | 6 mo | Cases | n=24 | 16.7% | 54.2% | 0.43 |
|  |  | Controls | n=14 | 35.7% | 50% |  |

| **Marker** | **Timepoint** |  | **# subjects** | **Increase** | **Decrease** | **P value** |
| --- | --- | --- | --- | --- | --- | --- |
| IL-5 | 1 mo | Cases | n=26 | 26.9% | 26.9% | 0.89 |
|  |  | Controls | n=12 | 25% | 25% |  |
|  | 6 mo | Cases | n=24 | 25% | 16.7% | 0.46 |
|  |  | Controls | n=14 | 21.4% | 28.6% |  |
| IL-6 | 1 mo | Cases | n=26 | 42.3% | 46.2% | 0.36 |
|  |  | Controls | n=11 | 9.1% | 54.6% |  |
|  | 6 mo | Cases | n=24 | 29.2% | 54.2% | 0.08 |
|  |  | Controls | n=12 | 50% | 25% |  |
| IL-10 | 1 mo | Cases | n=26 | 19.2% | 19.2% | 0.96 |
|  |  | Controls | n=12 | 25% | 25% |  |
|  | 6 mo | Cases | n=24 | 25% | 29.2% | 0.49 |
|  |  | Controls | n=14 | 35.7% | 21.4% |  |
| IL-13 | 1 mo | Cases | n=26 | 34.6% | 34.6% | 0.72 |
|  |  | Controls | n=12 | 25% | 41.7% |  |
|  | 6 mo | Cases | n=24 | 16.7% | 41.7% | 0.39 |
|  |  | Controls | n=14 | 35.7% | 35.7% |  |
| IL-15 | 1 mo | Cases | n=26 | 23.1% | 46.2% | 0.22 |
|  |  | Controls | n=12 | 33.3% | 25% |  |
|  | 6 mo | Cases | n=24 | 12.5% | 58.3% | 0.11 |
|  |  | Controls | n=14 | 35.7% | 35.7% |  |
| IL-17 | 1 mo | Cases | n=26 | 15.4% | 15.3% | 0.67 |
|  |  | Controls | n=12 | 16.7% | 25% |  |
|  | 6 mo | Cases | n=24 | 8.3% | 12.5% | 0.67 |
|  |  | Controls | n=14 | 42.9% | 50% |  |
| MIG | 1 mo | Cases | n=26 | 30.8% | 34.6% | 0.37 |
|  |  | Controls | n=12 | 60% | 33.3% |  |
|  | 6 mo | Cases | n=24 | 45.8% | 29.2% | 0.77 |
|  |  | Controls | n=13 | 46.2% | 38.5% |  |

| **Marker** | **Timepoint** |  | **# subjects** | **Increase** | **Decrease** | **P value** |
| --- | --- | --- | --- | --- | --- | --- |
| VEGF | 1 mo | Cases | n=26 | 7.7% | 42.3% | 0.99 |
|  |  | Controls | n=12 | 25% | 50% |  |
|  | 6 mo | Cases | n=24 | 20.8% | 50% | 0.36 |
|  |  | Controls | n=13 | 23.1% | 30.8% |  |
